# An Evidence-Based Serious Game App for Public Education on Antibiotic Use and Antimicrobial Resistance: Protocol of a Randomized Controlled Trial

**DOI:** 10.2196/45833

**Published:** 2023-03-28

**Authors:** Zhilian Huang, Wern Ee Tang, Huiling Guo, Karthiga Natarajan, Tau Hong Lee, Tsin Wen Yeo, Angela Chow

**Affiliations:** 1 Infectious Diseases Research and Training Office National Centre for Infectious Diseases Singapore Singapore; 2 Department of Preventive and Population Medicine Tan Tock Seng Hospital Singapore Singapore; 3 Clinical Research Unit National Healthcare Group Polycllinics Singapore Singapore; 4 Antimicrobial Resistance Coordinating Office National Centre for Infectious Diseases Tan Tock Seng Hospital Singapore Singapore; 5 Infectious Diseases Lee Kong Chian School of Medicine Singapore Singapore; 6 Lee Kong Chian School of Medicine Singapore Singapore; 7 Saw Swee Hock School of Public Health National University Singapore Singapore Singapore

**Keywords:** antibiotic resistance, antibiotic use, app development, development, educational intervention, health education, public education, randomized controlled trial, serious games, user engagement, user satisfaction

## Abstract

**Background:**

The misuse and overuse of antibiotics contribute to the acceleration of antimicrobial resistance (AMR), but public knowledge on appropriate antibiotic use and AMR remained low despite ongoing health promotion efforts. App gamification has gained traction in recent years for health promotion and to affect change in health behaviors. Hence, we developed an evidence-based serious game app “SteWARdS Antibiotic Defence” to educate the public on appropriate antibiotic use and AMR and address knowledge gaps.

**Objective:**

We aim to evaluate the effectiveness of the “SteWARdS Antibiotic Defence” app in improving the knowledge of, attitude toward, and perception (KAP) of appropriate antibiotic use and AMR among the public. The primary objective is to assess the changes in KAP of antibiotic use and AMR in our participants, while the secondary objectives are to assess the extent of user engagement with the app and the level of user satisfaction in using the app.

**Methods:**

Our study is a parallel 2-armed randomized controlled trial with a 1:1 allocation. We plan to recruit 400 participants (patients or their caregivers) aged 18-65 years from government-funded primary care clinics in Singapore. Participants are randomized in blocks of 4 and into the intervention or control group. Participants in the intervention group are required to download the “SteWARdS Antibiotic Defence” app on their smartphones and complete a game quest within 2 weeks. Users will learn about appropriate antibiotic use and effective methods to recover from uncomplicated upper respiratory tract infections by interacting with the nonplayer characters and playing 3 minigames in the app. The control group will not receive any intervention.

**Results:**

The primary study outcome is the change in participants’ KAP toward antibiotic use and AMR 6-10 weeks post intervention or 6-10 weeks from baseline for the control group (web-based survey). We will also assess the knowledge level of participants immediately after the participant completes the game quest (in the app). The secondary study outcomes are the user engagement level (tracked by the app) and satisfaction level of playing the game (via the immediate postgame survey). The satisfaction survey will also collect participants’ feedback on the game app.

**Conclusions:**

Our proposed study provides a unique opportunity to assess the effectiveness of a serious game app in public health education. We anticipate possible ceiling effects and selection bias in our study and have planned to perform subgroup analyses to adjust for confounding factors. The app intervention will benefit a larger population if it is proven to be effective and acceptable to users.

**Trial Registration:**

ClinicalTrials.gov NCT05445414; https://clinicaltrials.gov/ct2/show/NCT05445414

**International Registered Report Identifier (IRRID):**

DERR1-10.2196/45833

## Introduction

### Background

Antimicrobial resistance (AMR) has been a long-standing public health threat since the 1940s [1]. The widespread use of antibiotics has accelerated the growth of antibiotic-resistant bacteria, which can cause infections that are fatal and resistant to treatment [1]. In 2019, 4.95 million and 1.29 million global deaths were associated with and attributable to bacterial AMR [2]. It is estimated that 10 million global deaths will be attributable to AMR annually if no action is taken to tackle the problem [3].

AMR infections are often costly to health systems due to the excess length of hospital stays, more outpatient visits, and increased use of intensive care and isolation units [4]. For example, a methicillin-resistant *Staphylococcus aureus* infection could result in an additional US $31,338 (~SGD $42,620) hospitalization cost per patient [5]. AMR infections can also incur societal costs in terms of loss in work productivity and the risk of further infections [6,7].

The misuse and overuse of antibiotics are major drivers of AMR [8]. One pertinent factor contributing to the misuse of antibiotics is the public’s knowledge deficit of the indication for and proper use of antibiotics [9]. This knowledge deficit often leads to unnecessary patient demand for antibiotics and subsequent misuse of antibiotics [10,11]. Knowledge deficit in antibiotic use and AMR was consistently present among populations worldwide [12]. A nationally representative survey conducted in 2021 on adult Singaporeans demonstrated the association between poor knowledge of antibiotics and inappropriate use [9]. The study also showed that younger adults with poor knowledge had a higher likelihood of misusing antibiotics when compared with older adults, highlighting the need to address knowledge deficits on antibiotic use among the younger population in Singapore to mitigate the problem of AMR in the community [9].

### Rationale for the Study

Traditional mass public educational efforts using brochures, posters, and advertisements are extensive in outreach but questionable in improving the general public’s knowledge on appropriate antibiotic use and AMR [13,14]. In recent years, gamification—the use of game-playing elements to encourage the use of services—has gained attention as a novel strategy to affect change in health behaviors [15,16]. Increased user engagement can potentially lead to higher knowledge gains and retention for sustained behavioral change. Gamification was found to improve the knowledge of AMR. For example, a study found that the E-bug student website significantly increased the knowledge of appropriate antibiotic use among schoolchildren in the United Kingdom [17]. Another study using an web-based board game also found significant improvement in medical students’ knowledge of AMR immediately and 1 month post intervention [16]. These proof-of-concept studies support the effectiveness of gamification in increasing knowledge on appropriate antibiotic use among young people, which should be strengthened with randomized controlled trials along with other segments of the population and age groups.

### Hypothesis and Objectives

The public has low awareness of the gravity of AMR as many people perceive AMR as a nonimminent life-threatening problem for themselves [18]. We expect serious games to increase user engagement in learning, which leads to short- and long-term improvements in knowledge of, attitude toward, and perception (KAP) of the appropriate use of antibiotics. Leveraging digital technology to increase the awareness of AMR and the knowledge of appropriate antibiotic use is timely as Singapore develops into a digitalized nation. Hence, we have developed an evidence-based serious game app, “SteWARdS Antibiotic Defence,” to educate the public on appropriate antibiotic use and AMR. Users will learn about antibiotic use and AMR through the minigames and the bite-sized information released from the app.

We aim to evaluate the effectiveness of the “SteWARdS Antibiotic Defence” app in improving the KAP of appropriate antibiotic use and AMR among the public in Singapore. The primary objective is to compare the changes in KAP of antibiotic use and AMR among the app users with the control group. The secondary objectives are to assess (1) the extent of user engagement with the app by evaluating the users’ average screen time per day and (2) the level of user satisfaction in using the app for learning through a user satisfaction survey.

## Methods

### Study Design

**Figure 1:**
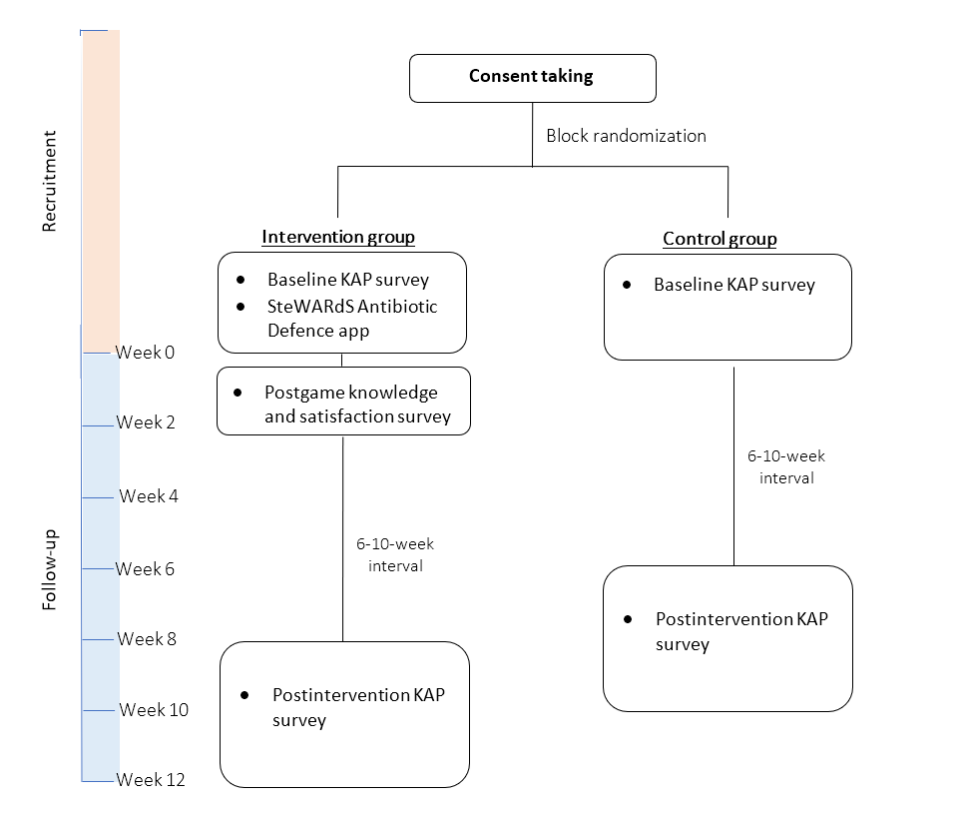
Trial schedule. KAP: knowledge of, attitude toward, and perception.

Our study is a parallel 2-armed randomized controlled trial (RCT) assessing the effectiveness of a serious game app in increasing the knowledge and changing the attitudes and perceptions about appropriate antibiotic use and AMR among the public. Participants randomized to the intervention group will be asked to download and complete the quest in the “SteWARdS Antibiotic Defence” app within 2 weeks on their smartphones. “SteWARdS Antibiotic Defence” is an evidence-based serious game app developed by the study team. The app will deliver bite-sized information on (1) the management of upper respiratory tract infections (URTIs), (2) appropriate antibiotic use, and (3) development of AMR to users as they complete the game quest (refer to the section on “Serious Game App Intervention”). Participants randomized to the control group will not be required to download the app. All participants will be followed up for 6-10 weeks to assess the effects of short-term knowledge retention. The design of the trial is presented in Figure 1.

### Recruitment

#### Study Setting

We will recruit patients or their caregivers from government-funded primary care clinics (ie, polyclinics) in Singapore. These polyclinics manage 20% of Singapore’s primary health care needs by providing subsidized services such as medical treatment for acute and chronic conditions, vaccinations, and health education [19]. The conditions seen at the polyclinics are acute and are managed outside the health care setting (eg, at home) after consultation with a health care provider. Hence, the intervention will occur outside the health care setting.

#### Inclusion and Exclusion Criteria

Participants must be older than 18 years, be a visitor of National Healthcare Group (NHG) polyclinic, have access to an Android smartphone, and be able to comprehend the English language to be eligible for the study. Those who are unable to install Android app, not proficient with smartphone apps, and have visual or cognitive impairment will be excluded from the study.

#### Randomization and Blinding

Participants will be block randomized into the intervention or control group (4 participants per block) by drawing lots from a box. They will not be blinded from the randomization as the study team has to explain the study to them. Only participants assigned to the intervention group have access to the serious game app.

### Serious Game App Intervention

#### App Development

The “SteWARdS Antibiotic Defence” app was developed in collaboration with Temasek Polytechnic, a public tertiary institution in Singapore. The study intervention involves the completion of a quest in the “SteWARdS Antibiotic Defence” app. Participants are required to respond to 11 baseline knowledge items before they can begin the game quest. The game quest involves passing all levels of 2 minigames (ie, Tower Defence and Match3), while the third game (ie, Endless Runner) allows participants unfamiliar with the game mechanics to earn extra coins to complete the intervention within the stipulated time frame.

Completing the baseline knowledge items is a prerequisite to proceed to the minigames, while completing the postgame knowledge items will fulfill the app intervention. The app will release bite-sized messages to reinforce concepts on the prevention and management of URTI and the appropriate use of antibiotics and AMR through gameplay and interaction with the nonplayer characters in the app. Participants are given only 3 “lives” daily to complete the Tower Defence and Match3 levels. Hence, the game completion may take slightly longer if the participant fails the levels more than 3 times in a day.

#### Evidence-Based Content

Many serious game apps intended for improving user knowledge failed to consider the cognitive process of learning, which limited the effectiveness of the intended outcome of the app. Therefore, we used Bloom’s revised taxonomy [20] as a guide to design the game mechanics. Concepts such as “antibiotics are effective for bacteria and not viruses” are reinforced repeatedly in the minigames to enhance memory. Users have to apply the concepts they learn to advance the game levels. For example, users must eliminate bacteria with the antibiotic turret and viruses with the symptomatic relief medication turret in the Tower Defence game and use the correct weapon to shoot the correct organism in the Endless Runner game. Users will also experience the “cost” of AMR by realizing that “misusing” antibiotic turrets in the earlier levels will drain their resources for tackling the superbug in the final level. Feedback (whether users respond to the items correctly or not) will be provided for the postgame knowledge survey to help users evaluate the concepts they learn from the game quest.

We derived the educational content in the app from a rigorous review of knowledge gaps [9,12,21], antibiotic guidance [22], and inputs from health care professionals. The in-app messages were also adapted to the local context to ensure their relevance to users.

#### Game Components

##### Overview

The app was designed with gamification elements to enhance user engagement and the learning process [23]. Gamification was achieved through clear rules, goal-oriented challenges, and rapid feedback. Users learn about appropriate antibiotic use and effective methods to recover from uncomplicated URTIs by interacting with the nonplayer characters and playing the minigames in the app. The 3 minigames are as follows.

##### Tower Defence

Players are required to build turrets (types of medication) to defend their fort (immunity bar) against enemies (bacteria or viruses) and have to cross 9 levels to complete this game. Players have to choose the appropriate turrets and strategically place them at appropriate locations to effectively target their enemies.

**Figure 2:**
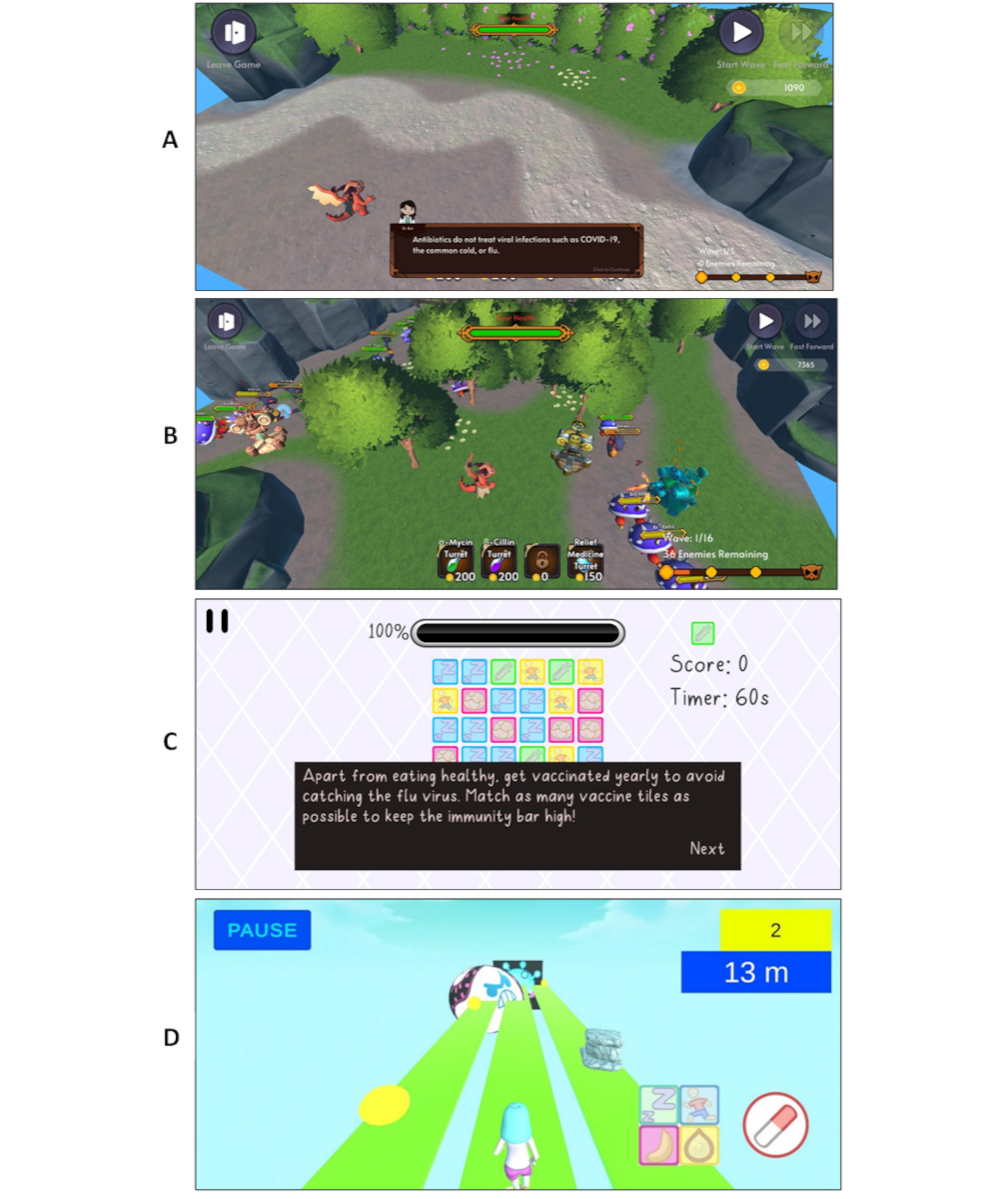
(A) Scene in the Tower Defence game with bite-sized educational messages. (B) Scene in the Tower Defence game with α-bac, β-bac, and viruses. (C) Scene in the Match3 game with messages for healthy living. (D) Scene in the Endless Runner game. The round icon represents a bacterium.

The first 2 levels teach players that antibiotics are effective against bacteria. Players are encouraged to buy and build antibiotic turrets to survive the onslaught of bacteria enemies (Figure 2A). The next 2 levels build on this knowledge by introducing the concept that antibiotics are bacteria specific. There will be 2 different types of bacteria (α-bac and β-bac), and players must build antibiotic turrets (α-mycin and β-cillin) specific to each bacteria enemy to clear the level (Figure 2B).

Levels 5 and 6 educate users that antibiotics are not effective against viruses. New enemies (viruses) are introduced, and players must buy immunity turrets to attack the viruses. To further instill this concept within players, bacteria and virus enemies attack simultaneously in levels 7 and 8, and players need to select the appropriate antibiotic and immunity turrets to complete the levels.

The last level educates players on antibiotic resistance by introducing multidrug-resistant organisms. Players have to invest in an expensive antibiotic turret effective against the multidrug-resistant organisms to complete this level.

The game difficulty increases with the levels, while players must be mindful of the coins they spend when purchasing and upgrading the necessary turrets. The immunity levels of the players are affected when too many enemies reach the end point. Players lose the level when their immunity bar falls to 0.

##### Match3

Match3 attempts to educate players on good health habits. Level 1 encourages participants to get a healthy amount of sleep. Level 2 focuses on the importance of a nutritious diet. The third level emphasizes on immunization, and the last level urges participants to care for their health in a holistic manner. Users are required to match 3 or more tiles within a specific time limit to successfully clear each level (Figure 2C).

##### Endless Runner

This game aims to reinforce the message that antibiotics are only effective against bacteria and not viruses. The player has to dodge obstacles and collect coins while running. An antibiotic gun is effective in shooting away bacteria obstacles, while an immunity gun can be used to shoot virus obstacles. This game also allows players to collect coins for use in the Tower Defence game in an engaging manner (Figure 2D).

### Study Procedures

We will randomize participants who are eligible for the study into the control or intervention group and collect their personal details in a password-protected file. Their demographic information will be collected on FormSG, a trusted web-based form manager developed by the Singapore government to collect survey data securely. We will require all participants to complete a 10-minute baseline KAP survey. For participants randomized into the intervention group, a study team member will assist participants in installing the “SteWARdS Antibiotic Defence” app on their mobile phones and provide further instructions regarding the game quest. Participants will need to complete the game quest in the app within 2 weeks, after which they will need to complete the postgame knowledge and an app satisfaction survey to complete the intervention. Participants will be contacted 6-10 weeks after completing the game quest and be asked to complete a 10-minute postintervention KAP survey.

Control group participants will complete a 10-minute baseline KAP survey and be contacted 6-10 weeks after recruitment to complete another 10-minute KAP survey. Participants will receive personalized messages via WhatsApp with a FormSG survey link and instructions to complete the postintervention survey during the study follow-up. We will provide reimbursement to all participants for their participation in the study. Reimbursement is structured according to behavioral economics principles to motivate participants to complete the intervention [24]. For example, reimbursement is divided over smaller amounts and is tiered to a higher value as the participant completes tasks toward the completion of the study. If the participant withdraws from the study, he or she will be asked to uninstall the app and will not be further contacted other than for reimbursement. The data collected prior to study withdrawal will be used for intention-to-treat analysis.

### Outcome Measures

#### Primary Outcomes

The primary outcome is the change in participants’ KAP toward antibiotics use and AMR 6-10 weeks post intervention from the baseline, measured by the KAP survey. We developed the KAP survey by assessing the literature [9,21,25] and adapting questions from the World Health Organization’s multicountry public awareness survey on antibiotic resistance [12]. Our KAP survey comprises 11 knowledge items with binary responses and 14 three-point Likert-scale items assessing participants’ attitudes and perceptions on antibiotics use and AMR (Table 1). We reverse-scored a few knowledge items in the postgame and postintervention surveys to reduce memory effects on knowledge gains.

**Table 1:** Knowledge, attitudes, and perception survey items.

Item number			Items		Response options
**Knowledge items**					True and false
	1	If bacteria are resistant to antibiotics, it can be very difficult or impossible to treat the infections they cause.			
	2	It is okay to buy the same antibiotics or request for them from a doctor, if they had helped you get better previously when you had the same symptoms.			
	3	It is okay to use antibiotics that were given to a friend or family member, as long as they were used to treat the same illness.			
	4	I can stop my antibiotics course when I start feeling better.			
	5	Antibiotic resistance is an issue that could affect me or my family.			
	6	Antibiotic resistance is only a problem for people who take antibiotics regularly.			
	7	Many infections are becoming increasingly resistant to antibiotics treatment.			
	8	Antibiotic resistance is an issue in other countries and in Singapore.			
	9	Antibiotic resistance occurs when your body becomes resistant to antibiotics and antibiotics no longer work as well.			
	10	Bacteria which are resistant to antibiotics can spread from person to person.			
	11	Antibiotic-resistant infections could make medical procedures like surgery, organ transplant, and cancer treatment much more dangerous.			
**Statements on attitudes and perceptions**					Agree, disagree, and unsure
	12	I feel that there is no harm in taking antibiotics.			
	13	I need antibiotics to help me to recover faster from the common cold and flu.			
	14	I need antibiotics to help me to recover from serious symptoms of the common cold and flu.			
	15	I need antibiotics if I continue to have flu symptoms after 2 weeks.			
	16	I will keep leftover antibiotics for future use if I have similar symptoms.			
	17	I would stop my course of antibiotics if I am concurrently using alternative remedy (eg, Traditional Chinese Medicine, Ayurveda medicine, Jamu).			
	18	I am scared of getting antibiotic resistance infections.			
	19	I will see another doctor if my doctor does not give me antibiotics.			
	20	How I use antibiotics does not affect my chances of getting antibiotic resistance infections.			
	21	How I use antibiotics does not affect other people’s chance of getting antibiotic resistance infections.			
	22	I normally keep antibiotic stocks at home in case of emergency.			
	23	If my family member is sick, I will usually give my antibiotics to them.			
	24	I normally stop taking antibiotics when I start feeling better.			
	25	I will take leftover antibiotics when I think I need them.			

#### Secondary Outcomes

The secondary outcomes are the assessment of the user engagement level (tracked by time spent on the app) and satisfaction level of playing the game (via the immediate postintervention survey). The satisfaction survey will also collect participants’ feedback on the game app.

### Sample Size

Based on 2 meta-analyses, the effect size of serious games on improving the knowledge of chronic disease management among young people is 0.361 (95% CI 0.098-0.624) [26] and healthy lifestyle promotion in general is 0.334 (95% CI 0.260-0.407) [27]. Using the smaller 2-tailed effect size of 0.334 with a 1:1 allocation ratio, a minimum sample size of 142 per group is required to detect significant changes in knowledge at a power of 80% and an α level of .05. Assuming an attrition rate of 30%, we will require a sample size of 200 participants per group for the study.

### Statistical Analysis

We will analyze the changes in the KAP scores with multivariable linear regression models and app satisfaction levels among intervention group participants with descriptive analyses. We will also conduct a post hoc power analysis to ensure that the sample size used in our study is adequately powered to detect a significant change.

### Ethical Considerations

This study was approved by the NHG Domain Specific Review Board in Singapore (National Healthcare Group Domain Specific Review Board Ref: 2022/00479).

## Results

We plan to recruit 400 participants for the study by April 2023. Results are expected to be achieved by June 2023. The data analysis and manuscript are expected to be completed by September 2023.

## Discussion

### Overview

Our protocol describes an upcoming RCT to evaluate the effectiveness of an evidence-based serious game app in changing the KAP of antibiotic use and AMR among the public. There is currently a paucity of RCTs on the effect of serious game apps on health promotion and health behavior change due to the complexity of evaluating such studies in the population. Many clinical teams have developed stand-alone apps to solve clinical problems, but these apps are often not evidence-based or validated with population-based studies. The myriad of stand-alone apps causes app fatigue and is often unsustainable and limited in its outreach.

The few serious game app studies are targeted at improving physicians’ antibiotic prescribing habits [28]. These studies had limited success in changing physicians’ prescribing habits and antibiotic use in the community, as patients can influence physicians’ prescribing decisions. Since antibiotic prescribing is multifactorial [29,30], it is imperative to concurrently target public awareness of appropriate antibiotic use to slow the progression of AMR. To our knowledge, this is the first RCT that assesses the effect of a serious game app for public education on antibiotic use and AMR. We developed the app with a rigorous review of evidence from the literature and inputs from clinicians, game design specialists, allied health professionals, academics, computer engineers, and members of the public. Therefore, this study will provide us with a rare opportunity to assess the effectiveness of serious game apps in public education to address the threats of AMR.

### Potential Limitations

Health promotion efforts to improve the public’s knowledge of antibiotics use are ongoing in Singapore via traditional mass media such as posters, advertisements, and billboards. These advertisements may contaminate and dilute the effects of our intervention but should not have a measurable impact on our study as the study period falls outside the national “antibiotic awareness week” campaign. Participants with good baseline knowledge may not see knowledge improvement due to the ceiling effect. We anticipate this problem to be minor, as a recent national study found that knowledge of good antibiotic use and AMR is poor among the population.

We will also ensure that only 1 adult from each household can participate in the study to minimize the possibility of contamination due to randomization (ie, 1 member is randomized into the control group and another into the intervention group). However, we would not be able to ensure that the recruited participant is the person completing the quest in the app, as family members (ie, children) may help to complete the game quest.

Older adults who are not technologically astute may also be excluded from our study or face difficulty in completing the game quest even if they meet the inclusion criteria. We will report problems with recruitment and follow-up and conduct subgroup analyses to assess outcomes between older and younger participants.

### Conclusions

Our proposed study provides a unique opportunity to assess the effectiveness of a serious game app in public health education. We may incorporate the “SteWARdS Antibiotic Defence” app as a minigame in an institutional health app platform to provide a sustainable learning resource for a larger population if the intervention is effective and acceptable to users. The findings of this study will also provide a reference for funders, researchers, policymakers, and organizations considering app-based educational interventions for the general population.

## Abbreviations

AMR: antimicrobial resistance
KAP: knowledge of, attitude toward, and perception
NHG: National Healthcare Group
RCT: randomized controlled trial
URTI: upper respiratory tract infection

## Data Availability

Data sharing is not applicable to this article as no data sets were generated or analyzed for this protocol.
